# Author Correction: Observation of a robust and active catalyst for hydrogen evolution under high current densities

**DOI:** 10.1038/s41467-023-36416-0

**Published:** 2023-02-06

**Authors:** Yudi Zhang, Kathryn E. Arpino, Qun Yang, Naoki Kikugawa, Dmitry A. Sokolov, Clifford W. Hicks, Jian Liu, Claudia Felser, Guowei Li

**Affiliations:** 1 CAS Key Laboratory of Magnetic Materials and Devices, and Zhejiang Province Key Laboratory of Magnetic Materials and Application Technology, Ningbo Institute of Materials Technology and Engineering, Chinese Academy of Sciences, Ningbo, 315201 China; 2 University of Chinese Academy of Sciences, 19A Yuquan Rd, Shijingshan District, Beijing, 100049 China; 3 Max Planck Institute for Chemical Physics of Solids, Nöthnitzer Strasse 40, 01187, Dresden, Germany; 4 National Institute for Materials Science (NIMS), Tsukuba, 305-0003 Japan; 5 Center for Advanced Solidification Technology, School of Materials Science and Engineering, Shanghai University, Shanghai, 200444 China

**Keywords:** Materials for energy and catalysis, Nanoscale materials, Hydrogen energy

Correction to: *Nature Communications* 10.1038/s41467-022-35464-2, published online 16 December 2022

In this article the affiliation details for Naoki Kikugawa were incorrectly given as ‘Max Planck Institute for Chemical Physics of Solids, Nöthnitzer Strasse 40, 01187, Dresden, Germany’ but should have been ‘National Institute for Materials Science (NIMS), Tsukuba, 305-0003, Japan’. The original article has been corrected.

