# Supplementary material for: Observation of a robust and active catalyst for hydrogen evolution under high current densities
Source: Nat Commun. 2022 Dec 16;13:7784. doi: 10.1038/s41467-022-35464-2 (PMC9758214; doi:10.1038/s41467-022-35464-2)
Supplement: Supplementary file 1 — Supplementary Information [file 41467_2022_35464_MOESM1_ESM.pdf]

Supporting information

Observation of a robust and active catalyst for hydrogen evolution under high current densities

Yudi Zhang[1,2], Kathryn E. Arpino[3], Qun Yang[3], Naoki Kikugawa[3], Dmitry Sokolov[3], Clifford W. Hicks[3], Jian Liu[1,2], Claudia Felser[3], Guowei Li[1,2]

1. CAS Key Laboratory of Magnetic Materials and Devices, and Zhejiang Province Key Laboratory of Magnetic Materials and Application Technology, Ningbo Institute of Materials Technology and Engineering, Chinese Academy of Sciences, Ningbo 315201, China
2. University of Chinese Academy of Sciences, 19 A Yuquan Rd, Shijingshan District, Beijing 100049, China
3. Max Planck Institute for Chemical Physics of Solids, Nöthnitzer Strasse 40, 01187, Dresden, Germany
4. Center for Advanced Solidification Technology, School of Materials Science and Engineering, Shanghai University, Shanghai 200444, China

Email: liguowei@nimte.ac.cn, claudia.felser@cpfs.mpg.de, liujian@shu.edu.cn

Density functional theory calculations.

Theoretical calculations were performed by using the Vienna ab initio simulation package (VASP) [1-3], and the generalized gradient approximation (GGA)[4] with the Perdew-Burke-Ernzerhof type exchange-correlation potential was adopted. For the H adsorption calculations, the (001) surface of SRO is chosen for the consideration of the dominating planes. The 2 × 2 were constructed by cleaving optimized bulk SRO in a six- layer-thick slab. The adsorption geometry of the Ru cluster at the SRO (001) surface is determined by exploring the most favorable binding energy. The differential adsorption energy of H adsorption is chosen to describe the stability of hydrogen according to the equation (1):

$$\Delta E_H = E(\text{catalyst} + \text{n H}) - E(\text{catalyst} + \text{(n-1) H}) - 1/2\ E(H_2)$$

Where E (catalyst + n H) is the total energy of catalysts (SRO or Ru_6/SRO) with the n hydrogen atoms adsorbed on the surface, E (catalyst + (n-1) H) is the total energy of SRO or Ru_6/SRO with (n-1) hydrogen atoms adsorbed on the surface, and E (H_2) is the total energy of the hydrogen molecule in the gas phase. This gives the binding energy of single H atom adsorption when n is equal to 1.

The $\triangle G_{H*}$ was used to predict the HER catalytic activity of the catalyst which can be obtained by following formulas (2) [5]:

$$\Delta G_{H*} = \Delta E_H + \Delta E_{ZPE} - T\Delta S_H$$

where ΔE_H is the adsorption energy, ΔE_{ZPE} is the zero-point energy (ZPE) difference, and ΔS_H is the entropy difference obtained from the entropy of H_2 gas under standard condition.

The zero-point energy $\triangle E_{ZPE}$ is obtained by equation (3)

$$\triangle E_{ZPE} = E_{ZPE}^{nH*} - E_{ZPE}^{(n-1)H*} - \frac{1}{2} E_{ZPE}^{H2}$$

where the E_{ZPE}^{nH*} and $E_{ZPE}^{(n-1)H*}$ denote the ZPE of n and n-1 adsorbed atomic hydrogens on the catalyst without the contribution of the catalyst, respectively. For the E_{ZPE}^{H2}, it is

the ZPE of H_2 which is calculated to be 274.4 meV in this work. In our calculation, E_{ZPE}^{H*} is calculated to be 178.3 meV for Ru2 site of Ru_6/SRO catalysts. Therefore, $\triangle E_{ZPE}$ is calculated to be 0.04 eV.

The entropy $\triangle S_H$ is obtained by equation (4)

$$\triangle \mathrm{S_H} \cong -\frac{1}{2}\mathrm{S_{H2}}$$

where the S_{H2} is the entropy of H_2 gas under the standard condition.

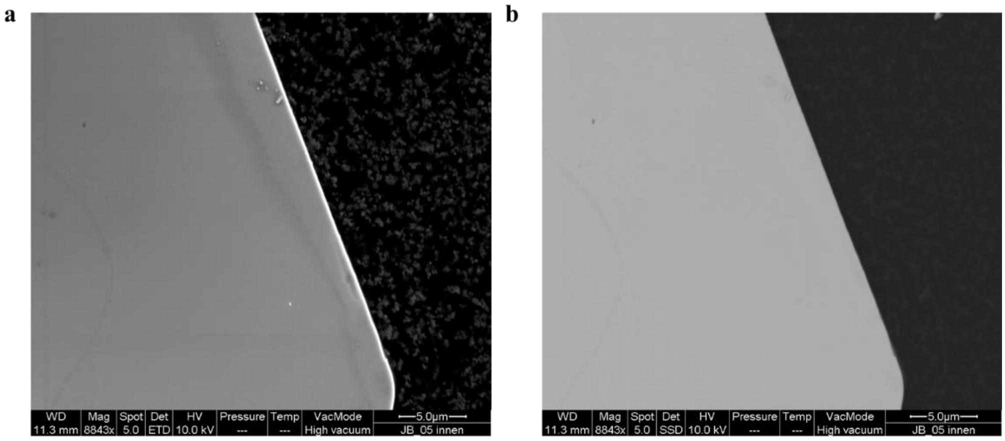

Fig. S1. SEM images of the fresh SRO single-crystal recorded with secondary electrons and b. backscattering electrons.

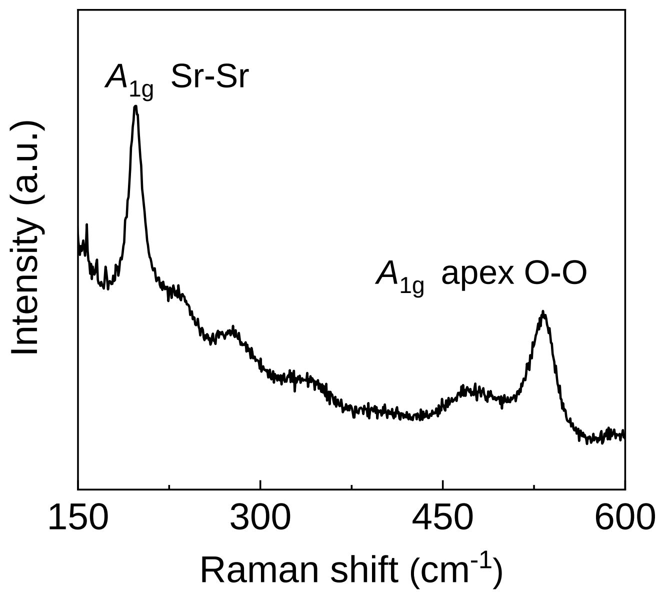

Fig. S2. Raman spectrum of the fresh SRO crystal.

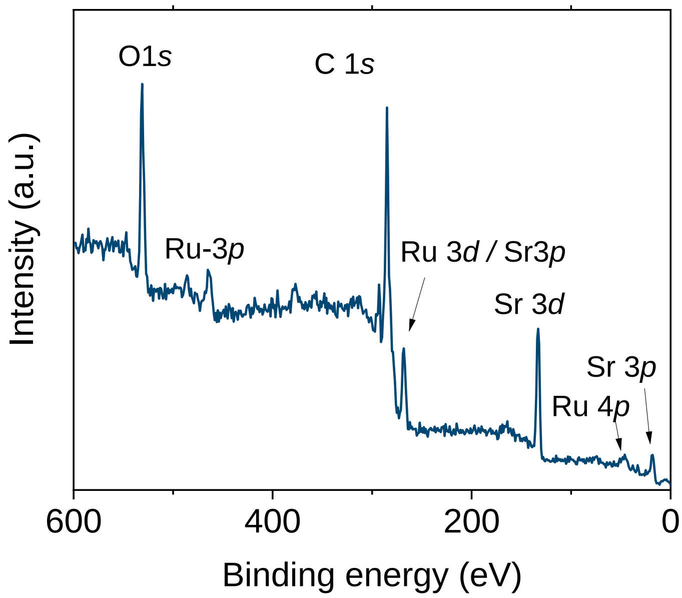

Fig S3. XPS survey spectrum of the fresh SRO before catalysis.

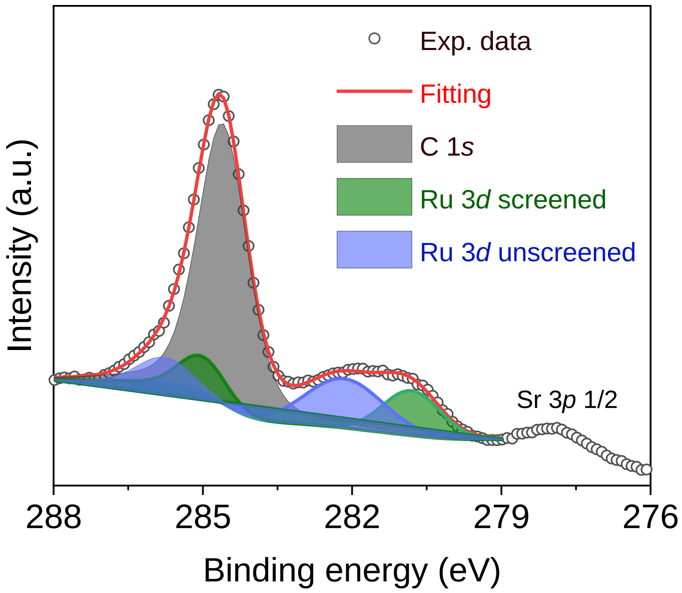

Fig. S4. XPS spectrum of the fresh SRO crystal taken at the Ru 3*d*.

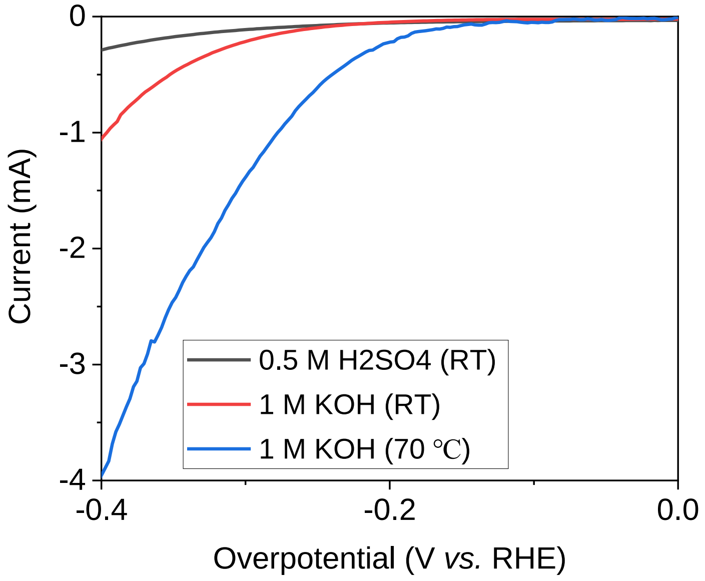

Fig. S5. LSV curves of Cu and silver paint at different measurement conditions (room temperature in 1 M KOH and 0.5 M H_2SO_4, and 70°C in 1 M KOH).

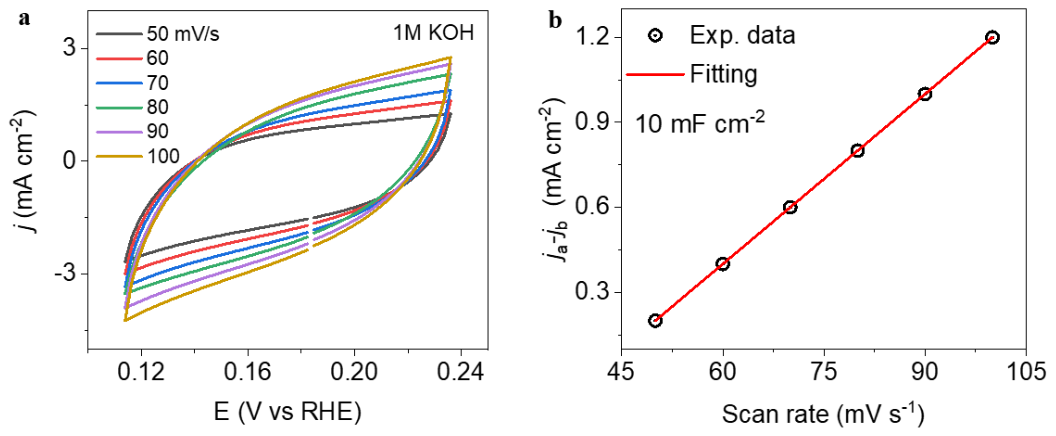

Fig. S6. a. CV curves of the activated SRO catalysts at different scan rates. **b.** double layer capacitance can be obtained by plotting the scan rate with current density at a given potential.

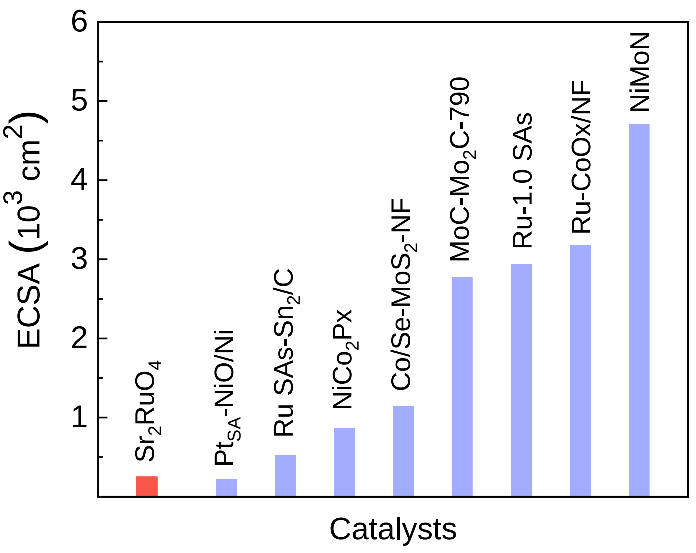

Fig. S7. Comparison of the ECSAs between the developed SRO/Ru catalysts with recently reported state-of-the-art catalysts.

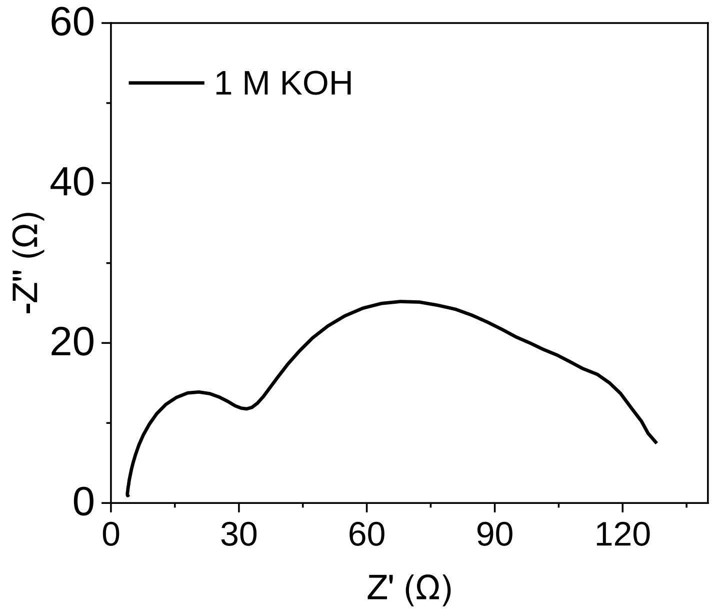

Fig S8. FRA spectrum of the activated SRO catalyst in 1M KOH electrolyte.

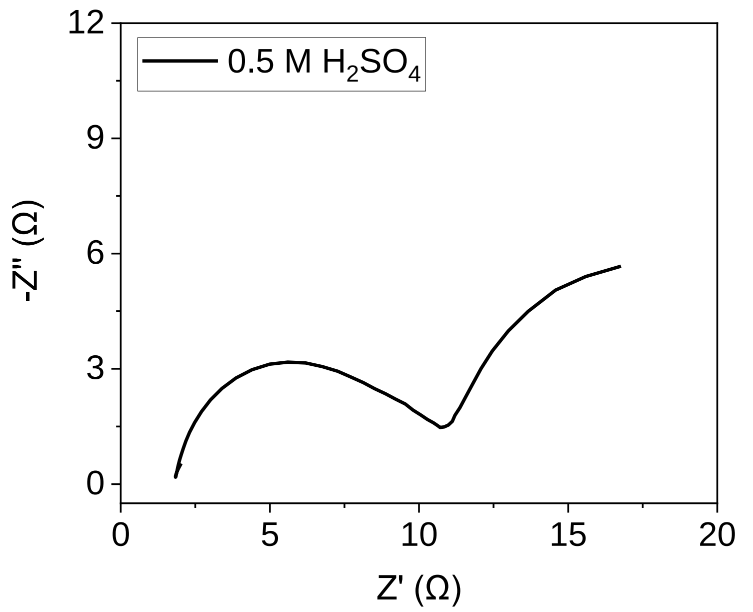

Fig. S9. FRA spectrum of the activated SRO catalyst in 0.5 M H_2SO_4 electrolyte.

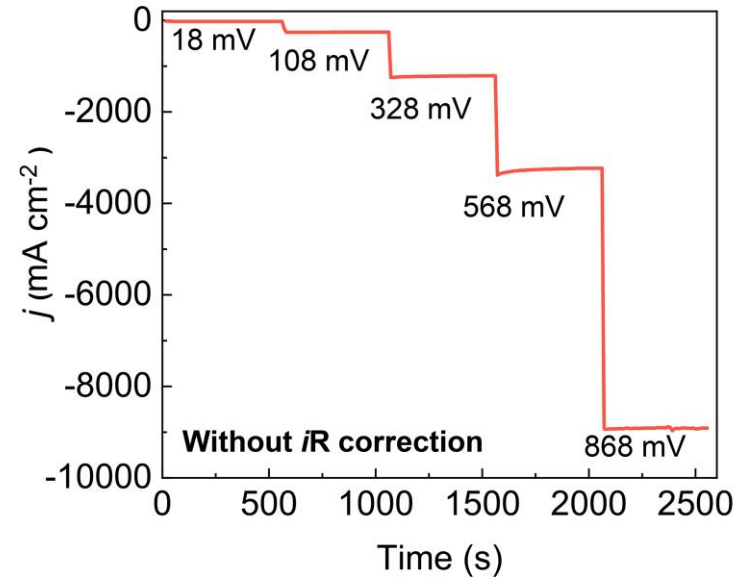

Fig. S10. The multicurrent process with a wide current density range from 20 to 9000 mA cm^{-2} without iR correction in 1M KOH.

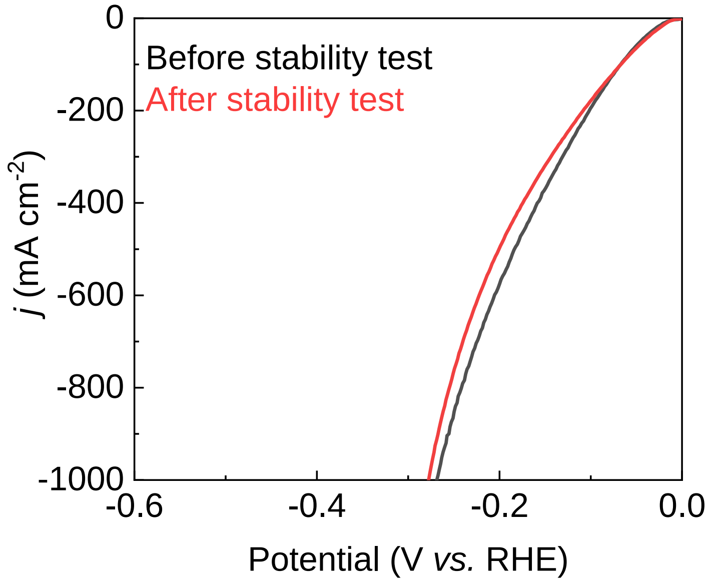

Fig. S11. Comparison of the LSV curves before and after the stability test in 1 M KOH electrolyte.

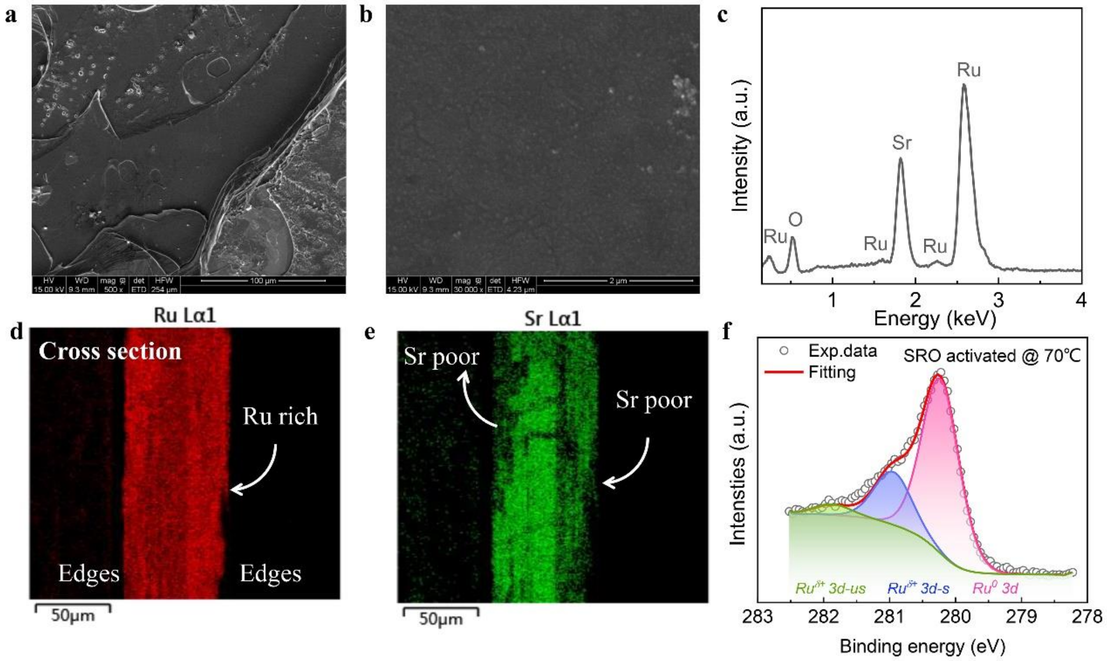

Fig. S12. a and b: SEM images of the SRO crystal surface after long-term testing at 70°C. c. EDS spectrum recorded at the same crystal after catalysis. Elemental mapping of the cross section d. Ru element, and e. Sr element. f. Ru 3d spectrum of the SRO crystal after catalysis.

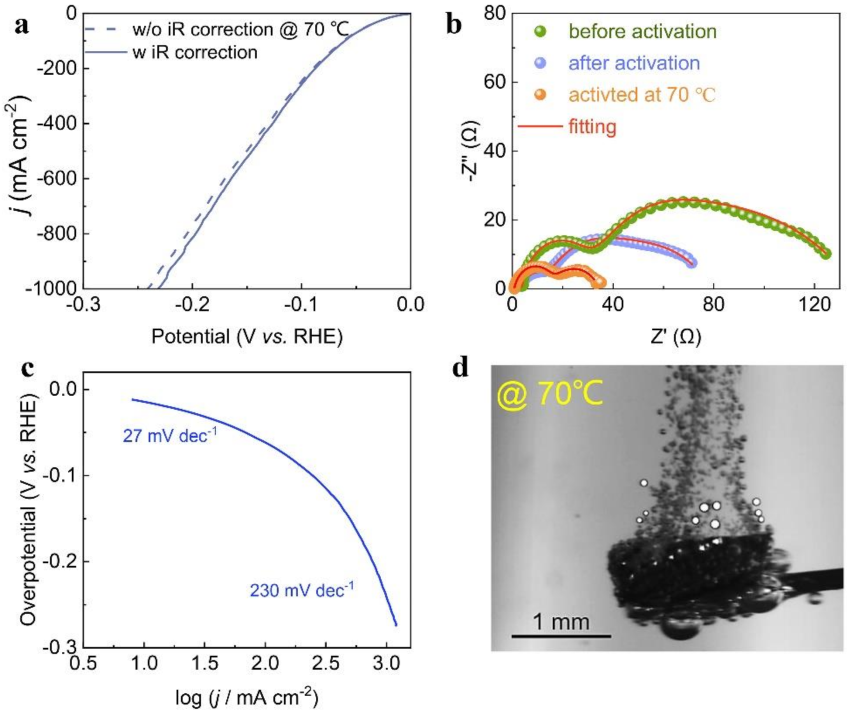

Fig. S13. (a) LSV curves of the SRO/Ru catalyst with (w) and without (w/o) considering the ohmic drop at 70℃. (b) Comparison of the Nyquist plots of SRO/Ru catalyst before and after HER testing at room temperature and 70℃. (c) Tafel slope analysis of the SRO/Ru at 70℃. (d) Hydrogen bubble release process at 70℃.

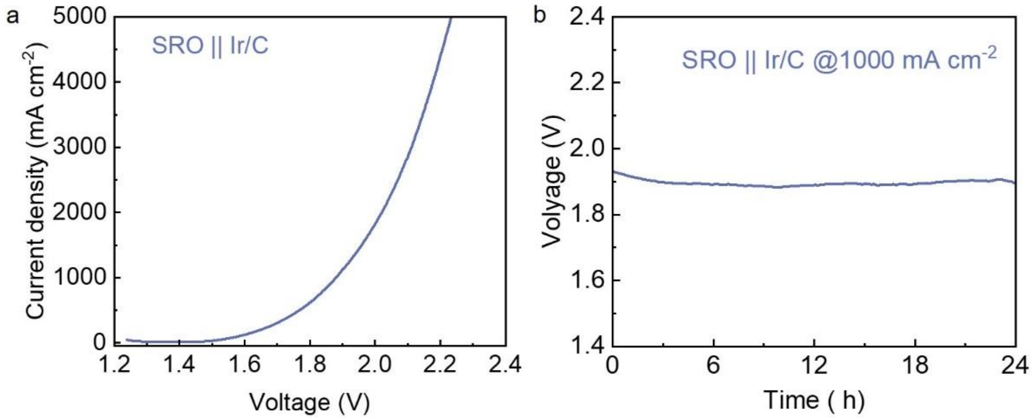

Fig. S14. (a) LSV curves of overall water electrolysis with the SRO as the cathode and commercial Ir/C as the anode in 1M KOH and the current density reaches 1000 mA cm^{-2}. (b) Long-term test of water electrolysis with SRO as the cathodes, and commercial Ir/C as the anode, indicating the high electrochemical stability.

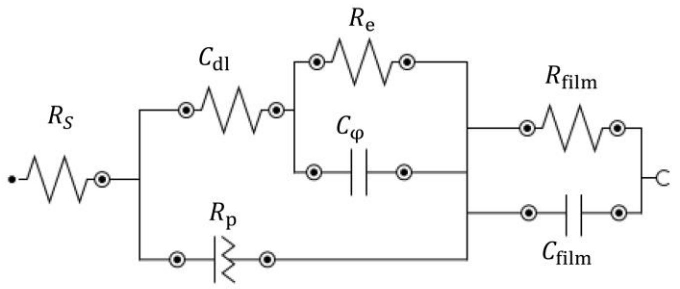

Fig. S15. The two-semi arcs model that used for fitting the impedance spectra. In the equivalent circuit, R_s represents the uncompensated solution resistance, C_{film} and R_{film} are related to the dielectric properties and the resistivity of the reconstructed Ru layer, while C_{dl} represents the double-layer capacitance. C_{φ} is the capacitance associated with the relaxation of the charge from the adsorbed reaction intermediate. R_p and R_e can be indexed to the kinetics of the interfacial charge transfer, with the former connected to the electrosorption process and the latter representing the resistivity as a result of the dielectric interlayer.

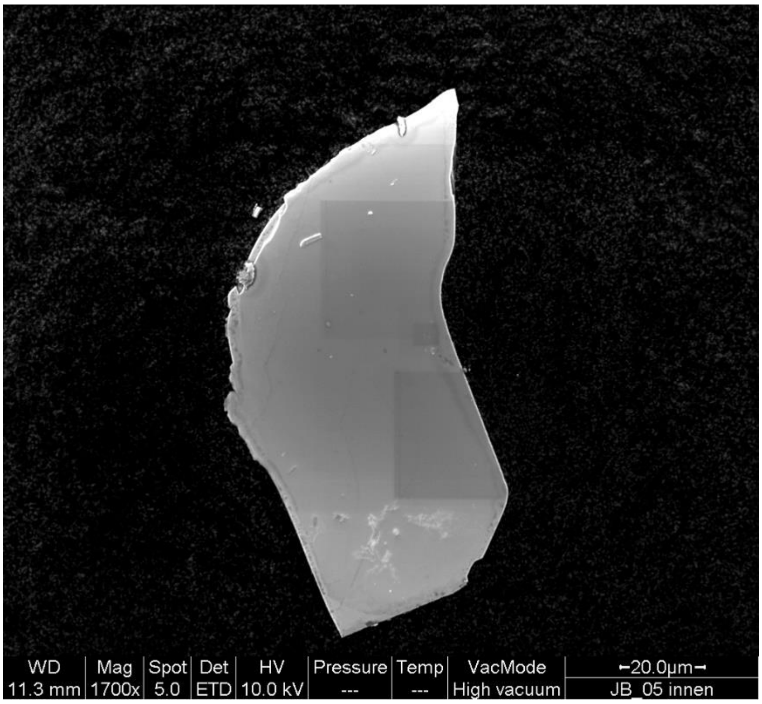

Fig. S16. SEM image of the SRO crystal surface before catalysis.

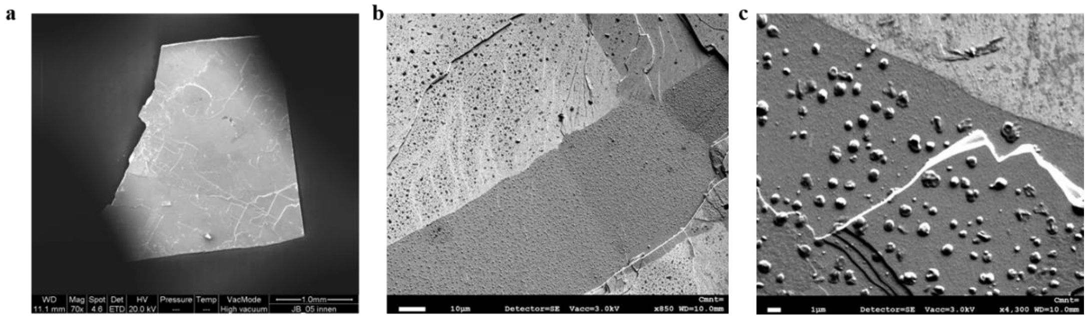

Fig. 17. SEM images of (001) SRO crystal surface after activation. One can see the increased roughness of the surface and the formation of numerous island-like particles.

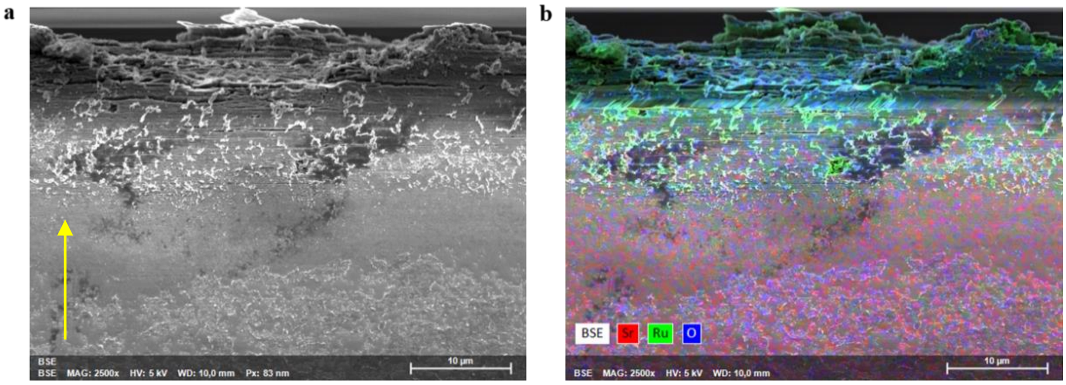

Fig. S18 a. SEM image of the activated SRO crystal after catalysis reaction. The arrow means along the *c* direction. **b.** Elemental mapping indicates the Ru element accumulation and the Sr element depletion at the top surface.

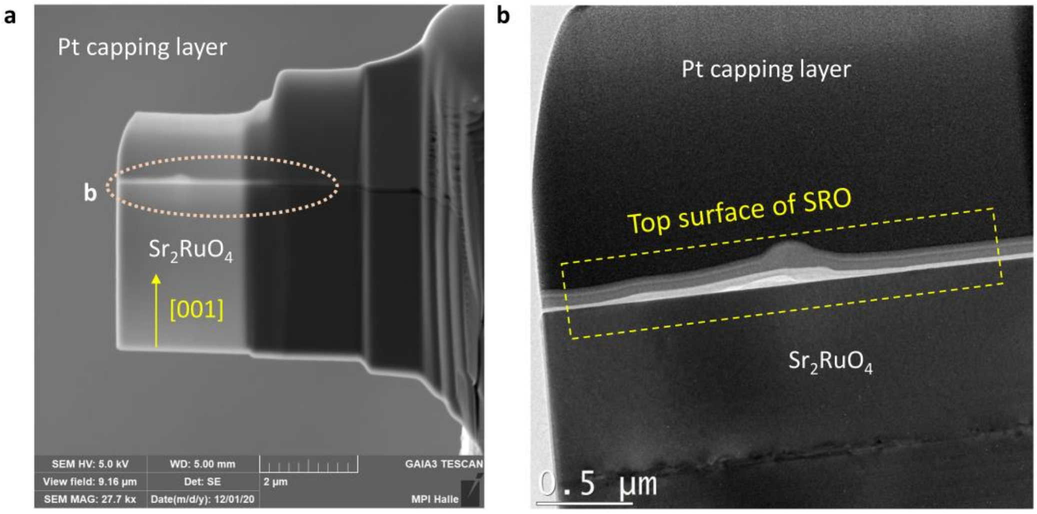

Fig. S19. TEM laminar of the SRO crystal after activation.

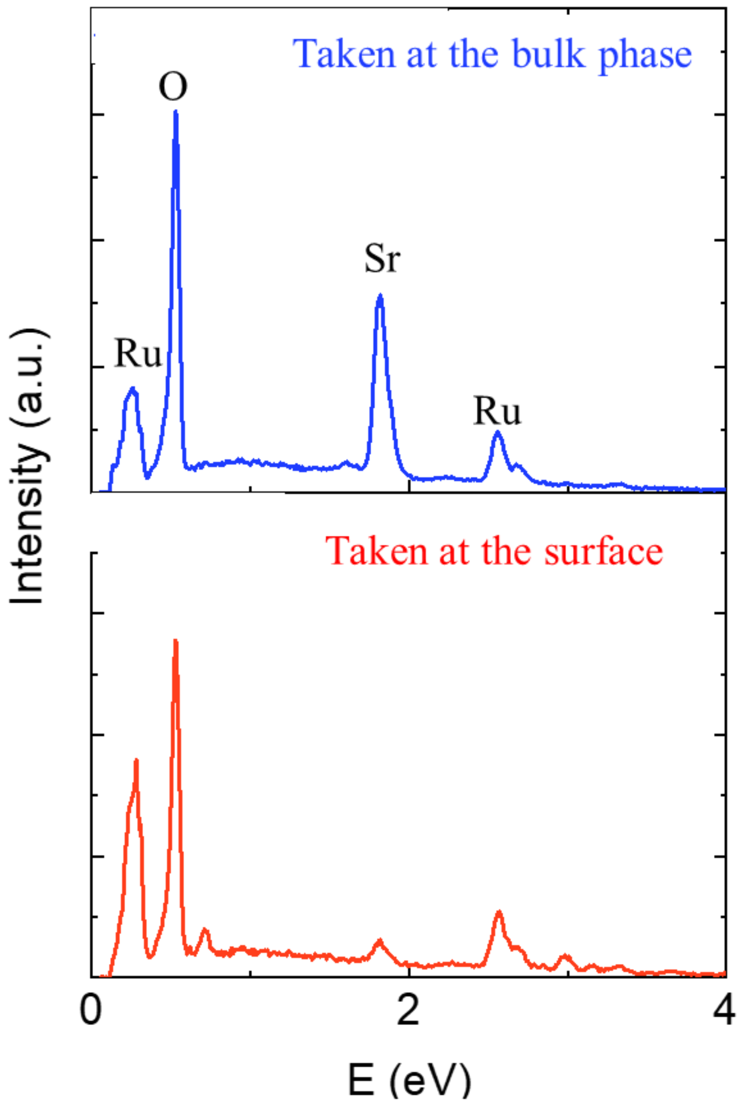

Fig. S20. EDS spectra taken from the surface and the internal bulk (recorded on the SEM of Fig. S18)

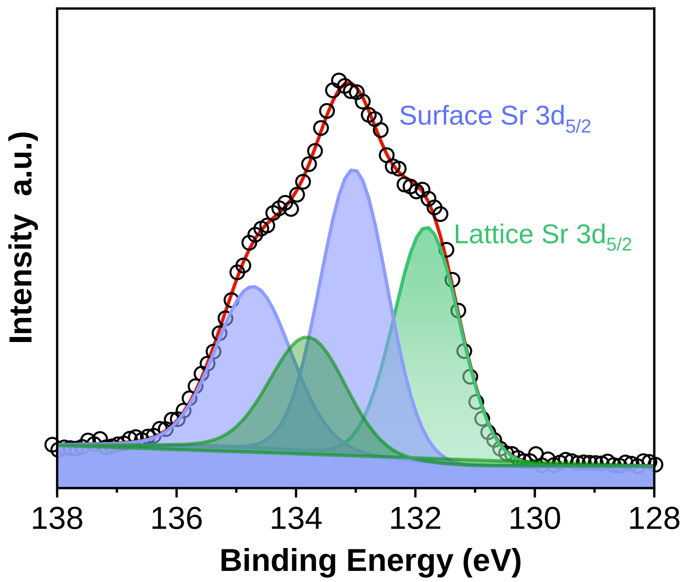

Fig. S21. XPS spectrum of Sr 3*d* peak after HER test. The component corresponds to the lattice Sr decreased significantly in comparison with the fresh crystal.

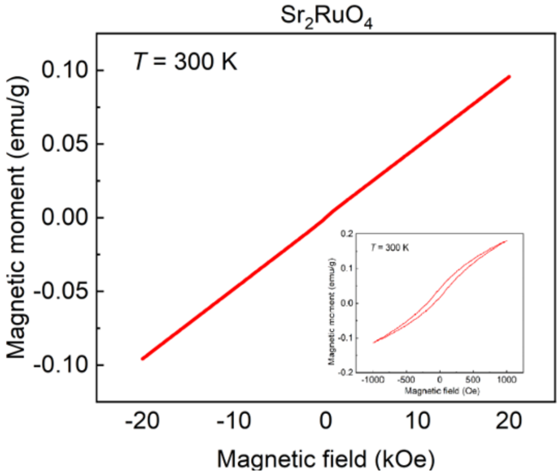

Fig. 22. Magnetic properties of the activated SRO crystal after catalysis. The main phase exhibits paramagnetic properties.

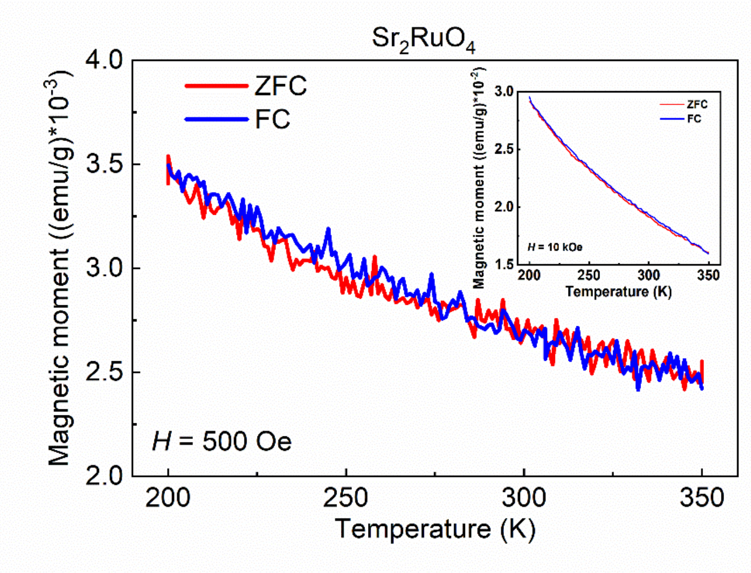

Fig. S23. ZFC-FC curves of the activated SRO single crystals. There is no magnetic transition up to 350 K.

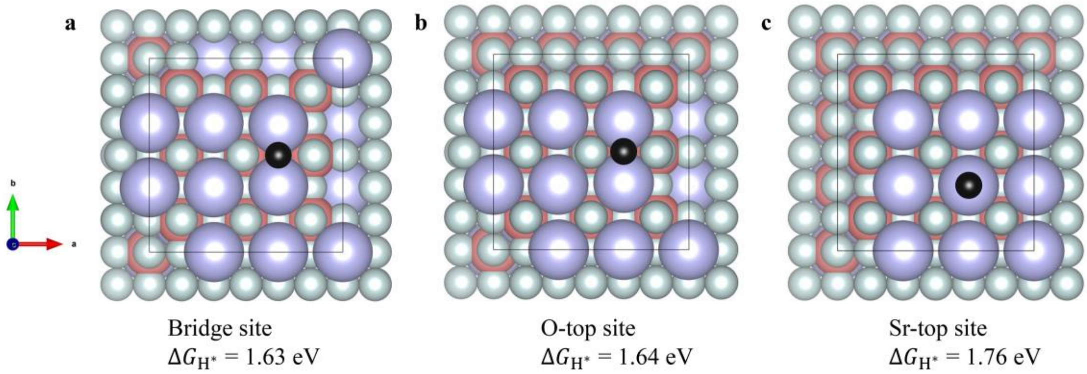

Fig. S24. The geometry of hydrogen adsorption at the pristine SRO (001) surfaces, **a.** bridge **b.** O-top site, and **c.** Sr-top site. Grey, pink, purple, and black balls represent O, Ru, Sr, and H atoms, respectively.

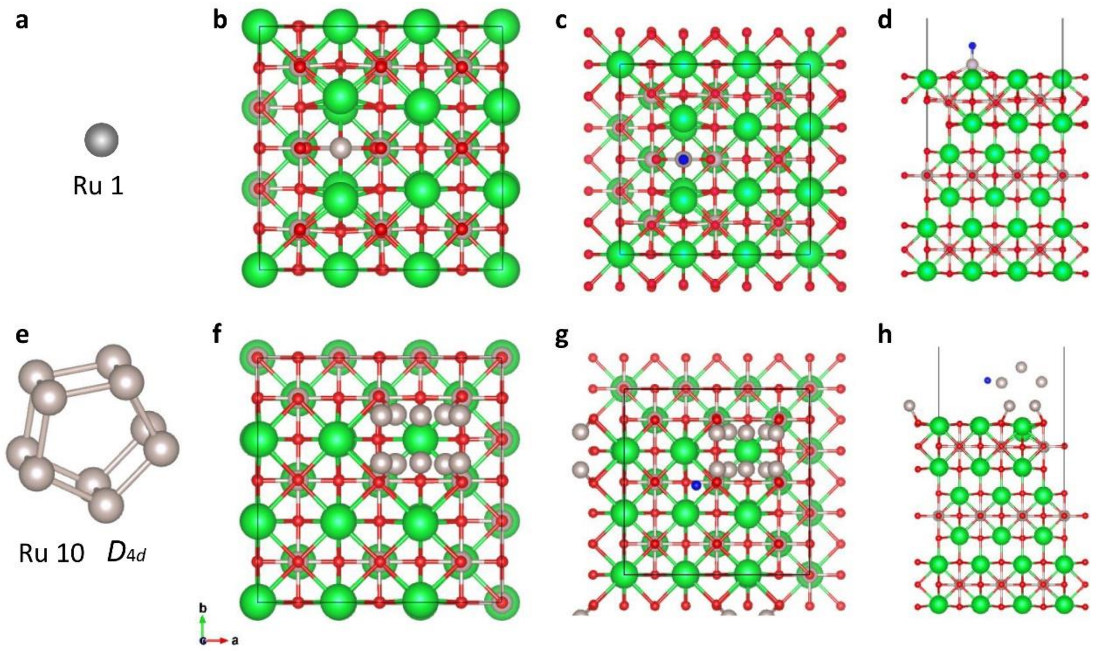

Fig. S25. Adsorption geometry of Ru 1 and Ru 10 clusters at the SRO (001) surface and the corresponding hydrogen adsorption configurations. Green, red, grey, and blue balls represent Sr, Ru, H, and Ru clusters, respectively. Ru cluster structures of a. Ru single atom, and b. Ru10 cluster. Adsorption geometry of b. Ru1/SRO, and f. Ru10/Ru. Top view of the hydrogen adsorption geometry for c. Ru1/SRO, and g. Ru10/SRO. d and h are the corresponding side view of hydrogen adsorption.

Materials	References
**SRO/Ru_6**	This work
**2H $Nb_{1.36}S_2$**	Ref. S6
**MoS_2 Low R_C**	Ref. S7
**MoS_2 Strained**	Ref. S8
**1T MoS_2**	Ref. S6
**CoPS**	Ref. S9
**MoS_2 vacancies**	Ref. S8
**CoP**	Ref. S10
**$[Mo_3S_{13}]^{2-}$ Low R_C**	Ref. S11
**Ru/C_3N_4**	Ref. S12

Tab. S1. Comparison of TOF values of the SRO catalysts with recently reported state-of-the-art catalysts.

Materials	Electrolyte solution	η@1000 mA cm^{-2} (mV)	Scan rate (mV/s)	References
**SRO/Ru_6**	0.5 M H_2SO_4	182	1	This work
**SRO/Ru_6**	1 M KOH	278	1	This work
**MoS_2/Mo_2C**	1 M KOH	224	5	Ref. S13
**Ta/TaS_2 MC**	0.5 M H_2SO_4	295	2	Ref. S14
**2H-$Nb_{1+x}S_2$**	0.5 M H_2SO_4	315	5	Ref. S6
**Fe-H_2**	1 M KOH	336	0.1	Ref. S15
**MoS_2**	0.5 M H_2SO_4	382	5	Ref. S16
**$Pd_4S/Pd_3P_{0.95}$**	1 M KOH	486	5	Ref. S17
**40% Pt/C**	0.5 M H_2SO_4	671	5	Ref. S16
**IrFe**	1 M KOH	850	2	Ref. S18

Tab. S2. Comparison of required overpotentials to reach the current density of 1000 mA cm^{-2} between the activated SRO catalyst and recently reported advanced catalysts.

Materials	Mass activities (A mg^{-1})	References
**SRO/Ru_6**	16.6	This work
**Pt_3Ni_2**	3.3	Ref. S19
**Ru WNO@C**	4.095	Ref. S20
**Ru cluster**	7.5	Ref. S21
**Ru HPC**	7.8	Ref. S22
**Ru-TA**	20	Ref. S23
**Ru-Pt**	23.1	Ref. S24

Tab. S3. Comparison of mass activities between activated SRO crystal and noble metal based HER catalysts.

	Top hollow	Top O	Top Sr
E_{slab+H} (eV)	-1265.36235323	-1265.36026350	-1265.23538619
E_{slab} (eV)	-1263.38745927	-1263.38745927	-1263.38745927
E_a (eV)	1.40510604	1.40719577	1.53207308

Tab. S4. Calculated energy of adsorbing hydrogen on the surface of SRO.

**Ru cluster**	**Magnetic property**	**Adsorption energy (Ru/SRO, eV)**	**Electron transfer (Ru to SRO, e^-)**	**ΔG_H*(eV)**
Ru 1	Paramagnetic	-6.13	0.21	1.86
Ru 6	Ferromagnetic	-6.90	0.29	0.12
Ru 10	Ferromagnetic	-8.57	0.24	1.69

Tab. S5. Structure and properties information for different Ru clusters and Ru/SRO interface.

	Site 1	Site 2
E_b (eV)	-0.35983708	-0.56798121
Gibbs (eV)	-0.12983708	-0.56798121
Charge transfer (eV)	0.2941403	0.1981467

Tab. S6. Calculated energy of adsorbing hydrogen on Ru_6 cluster.

Supplementary References

1. G. Kresse, J. Furthmüller, Efficiency of ab-initio total energy calculations for metals and semiconductors using a plane-wave basis set. *Comput. Mater. Sci.* **6**, 15-50 (1996).

2. G. Kresse, J. Furthmüller, Efficient iterative schemes for ab initio total-energy calculations using a plane-wave basis set. *Phys. Rev. B* **54**, 11169 (1996).

3. G. Kresse, D. Joubert, From ultrasoft pseudopotentials to the projector augmented-wave method. *Phys. Rev. B* **59**, 1758 (1999).

4. John P. Perdew, Kieron Burke, M. Ernzerhof, Generalized Gradient Approximation Made Simple. *Phys. Rev. Lett.* **77**, 3865-3868 (1996).

5. J. K. Nørskov *et al.*, Trends in the Exchange Current for Hydrogen Evolution. *J. Electrochem. Soc.* **152**, J23 (2005).

6. Yang J*, et al.* Ultrahigh-current-density niobium disulfide catalysts for hydrogen evolution. *Nat. Mater.* **18**, 1309-1314 (2019).

7. Voiry D*, et al.* The role of electronic coupling between substrate and 2D MoS_2 nanosheets in electrocatalytic production of hydrogen. *Nat. Mater.* **15**, 1003-1009 (2016).

8. Li H*, et al.* Corrigendum: Activating and optimizing MoS_2 basal planes for hydrogen evolution through the formation of strained sulphur vacancies. *Nat. Mater.* **15**, 364 (2016).

9. Wu T*, et al.* Crystallographic facet dependence of the hydrogen evolution reaction on CoPS: theory and experiments. *ACS Catal.* **8**, 1143-1152 (2018).

10. Hellstern TR, Benck JD, Kibsgaard J, Hahn C, Jaramillo TF. Engineering cobalt phosphide (CoP) thin film catalysts for enhanced hydrogen evolution activity on silicon photocathodes. *Adv. Energy Mater.* **6**, (2015).

11. Kibsgaard J, Jaramillo TF, Besenbacher F. Building an appropriate active-site motif into a hydrogen-evolution catalyst with thiomolybdate $[Mo_3S_{13}]^{2-}$ clusters. *Nat. Chem.* **6**, 248-253 (2014).

12. Li D, Liu Y, Liu Z, Yang J, Hu C, Feng L. Electrochemical hydrogen evolution

reaction efficiently catalyzed by Ru-N coupling in defect-rich Ru/g-C_3N_4 nanosheets. *J. Mater. Chem. A* **9**, 15019-15026 (2021).

13. Luo Y*, et al.* Morphology and surface chemistry engineering toward pH-universal catalysts for hydrogen evolution at high current density. *Nat. Commun.* **10**, 269 (2019).
14. Yu Q, Zhang Z, Qiu S. A Ta-TaS_2 monolith catalyst with robust and metallic interface for superior hydrogen evolution. *Nat. Commun.* **12**, 6051 (2021).
15. Zou X*, et al.* In situ generation of bifunctional, efficient Fe-based catalysts from mackinawite iron sulfide for water splitting. *Chem.* **4**, 1139-1152 (2018).
16. Zheng Z*, et al.* Boosting hydrogen evolution on MoS_2 via co-confining selenium in surface and cobalt in inner layer. *Nat. Commun.* **11**, 3315 (2020).
17. Zhang G*, et al.* Interfacial engineering to construct antioxidative $Pd_4S/Pd_3P_{0.95}$ heterostructure for robust hydrogen production at high current density. *Adv. Energy Mater.* **12**, (2022).
18. Jiang P*, et al.* Improving electrocatalytic activity of iridium for hydrogen evolution at high current densities above 1000 mA cm^{-2}. *Appl. Catal. B: Environ.* **258**, (2019).
19. Wang P*, et al.* Precise tuning in platinum-nickel/nickel sulfide interface nanowires for synergistic hydrogen evolution catalysis. *Nat. Commun.* **8**, 14580 (2017).
20. Zhang LN*, et al.* Cable-like Ru/WNO@C nanowires for simultaneous high-efficiency hydrogen evolution and low-energy consumption chlor-alkali electrolysis. *Energy Environ. Sci.* **12**, 2569-2580 (2019).
21. Wu YL*, et al.* Ordered macroporous superstructure of nitrogen-doped nanoporous carbon implanted with ultrafine Ru nanoclusters for efficient pH-universal hydrogen evolution reaction. *Adv. Mater.* **33**, e2006965 (2021).
22. Qiu T*, et al.* Highly exposed ruthenium-based electrocatalysts from bimetallic metal-organic frameworks for overall water splitting. *Nano Energy* **58**, 1-10 (2019).
23. Chen J, Wang H, Gong Y, Wang Y. Directly immobilizing a Ru-tannic acid

linkage coordination complex on carbon cloth: an efficient and ultrastable catalyst for the hydrogen evolution reaction. *J. Mater. Chem. A* **7**, 11038-11043 (2019).

24. Zhang L*, et al.* Atomic layer deposited Pt-Ru dual-metal dimers and identifying their active sites for hydrogen evolution reaction. *Nat. Commun.* **10**, 4936 (2019).
