# Supplementary material for: Observation of a robust and active catalyst for hydrogen evolution under high current densities
Source: Nat Commun. 2022 Dec 16;13:7784. doi: 10.1038/s41467-022-35464-2 (PMC9758214; doi:10.1038/s41467-022-35464-2)
Supplement: Supplementary file 3 — Description of Additional Supplementary Files [file 41467_2022_35464_MOESM3_ESM.pdf]

Description of Additional Supplementary Files

File Name: Supplementary Movie 1
Description: Hydrogen bubble size analysis and release kinetics at the surface of Ru_6/SRO crystal during a test time of 25 seconds (The operating current density is 1000 mA cm^{-2})

File Name: Supplementary Movie 2
Description: Hydrogen bubble size analysis and release kinetics at the surface of Pt electrode during a test time of 25 seconds (The operating current density is 1000 mA cm^{-2})
